# Left Atrial Reservoir Strain and Heart Failure Rehospitalization Burden in Acute Heart Failure With Atrial Fibrillation

**DOI:** 10.1111/echo.70616

**Published:** 2026-09-04

**Authors:** Jumpei Yamamoto, Keijiro Nakamura, Hiromasa Hayama, Yoshinari Enomoto, Masaya Yamamoto, Hidehiko Hara, Hisao Hara, Yukio Hiroi

**Affiliations:** ^1^ Division of Cardiovascular Medicine Toho University Medical Center Ohashi Hospital Ohashi, Meguro‐ku Tokyo Japan; ^2^ Department of Cardiology National Center for Global Health and Medicine, Japan Institute for Health Security Shinjuku‐ku Tokyo Japan

**Keywords:** atrial fibrillation, acute heart failure, echocardiography, left atrial strain, recurrent events

## Abstract

**Purpose:**

To examine associations of left atrial reservoir strain, diameter, and volume index with first rehospitalization and recurrent readmission burden in acute heart failure with atrial fibrillation.

**Methods:**

Among 716 enrolled patients, 691 with an available measurement formed the left atrial diameter analysis cohort; left atrial volume index (LAVI) and reservoir strain (LArs) were evaluated in a 320‐patient single‐center subgroup. Same‐day rehospitalization and death were classified as death‐first. Fine–Gray and negative‐binomial models examined first rehospitalization and recurrent readmission burden.

**Results:**

Of 691 patients, 189 had rehospitalization‐first, 118 death‐first, and 384 were censored. In the 320‐patient subgroup, lower LArs was associated with first rehospitalization (adjusted subdistribution hazard ratio [sHR] 0.920 per 1 percentage‐point increase, 95% confidence interval [CI] 0.870–0.973) and with a higher recurrent readmission rate (incidence rate ratio 0.877, *p* < 0.001); the first‐event association persisted after additional echocardiographic adjustment including left atrial diameter (sHR 0.928, 95% CI 0.876–0.984). Left atrial diameter was associated with rehospitalization‐first (sHR 1.025/mm, 95% CI 1.011–1.039) but not death‐first, whereas LAVI was associated with neither, including per standard deviation. With exploratory cutoffs, only combined abnormality was associated with first rehospitalization (sHR 2.12, 95% CI 1.16–3.85). Left atrial indices did not significantly improve discrimination.

**Conclusion:**

Lower LArs was associated with first heart failure rehospitalization and recurrent readmission burden, and remained associated after additional echocardiographic adjustment including left atrial diameter. Left atrial diameter was associated with first rehospitalization but LAVI was not; a general left atrial structural mechanism is not established. Incremental predictive value was limited; cohort‐derived cutoffs require external validation.

## Graphical abstract

This graphical abstract summarizes 716 patients with acute heart failure and atrial fibrillation. The left atrial diameter analysis included 691 patients (189 rehospitalization‐first, 118 death‐first, 384 censored); volume index and reservoir strain were available in a 320‐patient single‐center subgroup. Lower reservoir strain was associated with both first heart failure rehospitalization and recurrent readmission burden and remained associated after additional echocardiographic adjustment including left atrial diameter and volume index. Left atrial diameter was associated with first rehospitalization; volume index was associated with neither first rehospitalization nor recurrent readmission burden. Discrimination did not materially improve; cohort‐derived cutoffs require external validation.

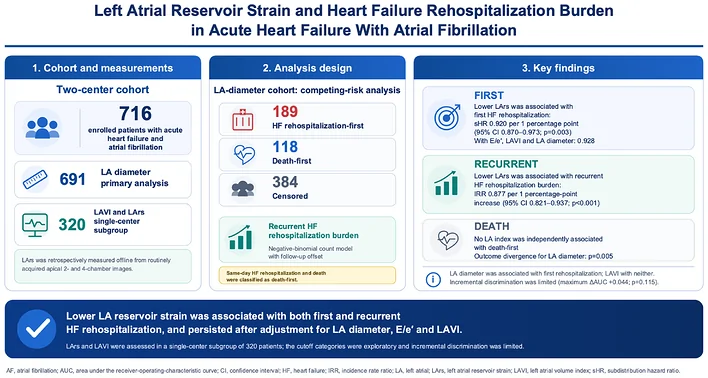

**Abbreviations**
AF: atrial fibrillation
AHF: acute heart failure
AUC: area under the receiver‐operating‐characteristic curve
CI: confidence interval
eGFR: estimated glomerular filtration rate
E/e′: ratio of early mitral inflow velocity to mitral annular early diastolic velocity
HF: heart failure
LA: left atrial
LArs: left atrial reservoir strain
LAVI: left atrial volume index
LVEF: left‐ventricular ejection fraction
MR: mitral regurgitation
NCGM: National Center for Global Health and Medicine
sHR: subdistribution hazard ratio

## 1 Introduction

Atrial fibrillation (AF) and heart failure (HF) often coexist, each worsening the other's prognosis; AF is especially common in patients admitted for acute heart failure (AHF) [1, 2]. Prognostic research in this setting has centered on mortality. Yet, HF rehospitalization occurs more often, accounts for much of the morbidity and cost, and is now viewed as a patient‐centered outcome in its own right [3]. Whether rehospitalization and death share the same predictors has rarely been tested directly, even though the two events may arise from different pathophysiology and call for different preventive strategies.

The left atrium (LA) sits at the center of the AF–HF interface [4]. LA enlargement reflects chronic remodeling under a sustained filling‐pressure load. LA diameter is only a one‐dimensional measure of a chamber that remodels asymmetrically, but it is recorded in almost every routine study; the preferred volumetric index, the LA volume index (LAVI), and LA reservoir strain (LArs) need dedicated volumetric or speckle‐tracking analysis and are far less widely available [5]. LArs captures functional impairment and tracks left‐ventricular filling pressure more closely than LA volume or the ratio of early transmitral inflow velocity to early diastolic mitral annular velocity (E/e′) [6]. Reduced LA function has been linked to adverse outcomes in HF [7, 8]. Our previous report focused on mortality in the NCGM cohort [9]; the present study addresses HF rehospitalization and recurrent burden. Less is known about whether LA size and LArs convey the same information for HF rehospitalization, for death‐first, and for recurrent readmission burden.

We therefore examined three questions in a two‐center cohort of patients hospitalized for AHF with AF: whether LArs is associated with first rehospitalization and with recurrent readmission burden independently of left atrial size and of E/e′; whether left atrial diameter, LAVI, and LArs contribute differently to these outcomes; and whether the markers associated with HF rehospitalization‐first and death‐first diverge.

### 1.1 Patients and Methods

We retrospectively studied consecutive patients aged ≥18 years admitted for AHF complicated by AF at two Japanese cardiovascular centers. Patients were followed from the index admission until death, last contact, or the end of observation.

The cohort comprised 396 patients from Toho University Ohashi Medical Center and 320 from the National Center for Global Health and Medicine (NCGM). Of the 716 enrolled patients, 25 without an available left atrial diameter measurement were excluded from analyses involving left atrial diameter, leaving 691 patients for the primary left atrial diameter analysis. LAVI and LArs were available only in the NCGM cohort and were analyzed in this 320‐patient cohort. Analyses used complete cases for the variables required in each model; no simple or multiple imputation or missing‐indicator method was applied, and model‐specific sample sizes are reported with each estimate.

### 1.2 Endpoints

Follow‐up began at the index admission. First HF rehospitalization and death were analyzed as competing first events, and patients without either event were censored at the last documented contact. Same‐day rehospitalization and death were classified as death‐first in the primary analysis, because such an admission may represent a terminal event rather than a readmission for decompensation, and as rehospitalization‐first in a sensitivity analysis. A further sensitivity analysis used cardiovascular rather than all‐cause death as the competing event. The total number of HF readmissions was available in the NCGM cohort and was used for the recurrent‐burden analysis.

### 1.3 Echocardiography

Transthoracic echocardiography was performed at admission using an Aplio400 or Artida ultrasound system (Toshiba Medical Systems, Otawara, Japan) according to standard M‐mode, two‐dimensional, and Doppler protocols. Routine echocardiographic measurements were obtained from the admission examination. Left atrial diameter was assessed using two‐dimensional echocardiography from the parasternal long‐axis view in all patients with an available measurement. Left‐ventricular volumes and left‐ventricular ejection fraction (LVEF) were obtained by the modified Simpson method from apical 2‐ and 4‐chamber views, and left atrial volume by the biplane area‐length method, indexed to body surface area as LAVI. The E/e′ ratio was derived from transmitral early diastolic E velocity at the mitral leaflet tips and septal e′ velocity by tissue Doppler at the septal mitral annulus, in line with chamber‐quantification recommendations [5]. Mitral regurgitation (MR) was graded semiquantitatively on the admission study at NCGM. Significant MR was defined as moderate or greater and was present in 100 of 320 patients; the remainder were classified as without significant MR.

In the NCGM cohort, LArs was retrospectively measured offline from routinely acquired apical 2‐ and 4‐chamber images with two‐dimensional speckle‐tracking software (AutoStrain, Image Arena; TOMTEC Imaging Systems, Unterschleissheim, Germany) [9, 10]. QRS onset served as the zero‐reference point; endocardial borders were traced automatically and corrected by hand when tracking was inadequate; and each view was analyzed over a single cardiac cycle at a frame rate >45. Mean LArs was taken as the absolute value averaged across the 2‐ and 4‐chamber views. As previously reported, strain analysis was performed by a single cardiologist blinded to clinical data. The mean heart rate during echocardiography was 86 ± 20 bpm, cross‐view heart‐rate agreement was good (intraclass correlation coefficient 0.82, 95% confidence interval [CI] 0.78–0.85), and intra‐observer reproducibility in 30 randomly selected patients was excellent (intraclass correlation coefficient 0.92, 95% CI 0.85–0.96) [9]. LArs in this cohort fell far below published normative values for healthy adults [11], which is the reason the cohort‐specific cutoffs described below were examined alongside published reference thresholds.

### 1.4 Statistical Analysis

Continuous variables are reported as median (interquartile range) and categorical variables as counts (percentages). Cumulative incidence functions were estimated under competing risks and compared with Gray's test. Associations with the cumulative incidence of HF rehospitalization were estimated using Fine–Gray subdistribution hazard models [12], and cause‐specific Cox models were fitted in parallel to examine etiologic contrasts and death‐first events [13]. The core adjustment set was fixed in advance on clinical grounds rather than by data‐driven selection: age and sex for demographic confounding; estimated glomerular filtration rate (eGFR) and hemoglobin for renal and anemia‐related vulnerability; LVEF for systolic function; natriuretic peptide for congestion severity; and study center for the two‐center left atrial diameter analysis. Because B‐type natriuretic peptide and N‐terminal pro–B‐type natriuretic peptide were measured at different centers, natriuretic peptide was entered as a within‐center standardized logarithmic value. Separate sensitivity models added hypertension and diabetes; replaced continuous eGFR with chronic kidney disease (eGFR <60 mL/min/1.73 m^2^); and, within the NCGM cohort, added body mass index and significant MR.

To test whether a predictor was differentially associated with rehospitalization‐first versus death‐first, we applied the Lunn–McNeil stacked‐data approach with a predictor‐by‐cause interaction and robust variance [14]. Continuous left atrial diameter and LArs were the primary analyses. Exploratory cohort‐derived cutoffs for left atrial diameter and LArs were selected at 365 days in the NCGM cohort by maximizing the Youden index from inverse‐probability‐of‐censoring‐weighted time‐dependent receiver‐operating‐characteristic curves that treated death‐first as a competing event. These cutoffs defined the exploratory structure–function categories; published reference thresholds (LArs <18%, reported to identify elevated left‐ventricular filling pressure [15]; LAVI >34 mL/m^2^ [5] and median‐based categories were examined as sensitivity analyses. Spearman correlations and variance inflation factors described the relation between left atrial size and LArs and the stability of models containing both. To compare the two size measures on a common scale, left atrial diameter and LAVI were also modeled per 1 standard deviation in the NCGM cohort, and departure from linearity was assessed for each measure with a natural spline (3 degrees of freedom) tested against the linear term by likelihood ratio, using the same framework applied to LArs. Recurrent readmission counts were modeled using negative binomial regression [16] with the logarithm of follow‐up time as an offset; because individual readmission dates were unavailable, this was a count‐burden analysis rather than a death‐adjusted recurrent‐event analysis. As a secondary analysis, incremental predictive performance was assessed using the time‐dependent area under the receiver‐operating‐characteristic curve (AUC), and Brier scores at 1 and 3 years. Sensitivity analyses evaluated alternative scaling, center and LVEF interactions, selected additional covariates, alternative competing‐event definitions, a 30‐day landmark, proportional‐hazards assumptions, and non‐linearity; details are provided in the Supporting Information. Patients in the NCGM cohort were compared with the Toho cohort using standardized mean differences. A two‐sided *p* < 0.05 was significant. Analyses were conducted in R 4.5.1 (R Foundation for Statistical Computing, Vienna, Austria).

## 2 Results

### 2.1 Patients and Events

Of 716 enrolled patients (mean age 79 years; 49% women; 82% on oral anticoagulants), 396 were enrolled at Toho University Ohashi Medical Center and 320 at NCGM. Left atrial diameter was available in 691 patients; the 25 patients without a measurement were from Toho, leaving 320 NCGM and 371 Toho patients in the left atrial diameter analysis cohort. Over a median follow‐up of 503 days (interquartile range, 84 to 979 days), 189 had HF rehospitalization‐first, 118 died first, and 384 were censored in this 691‐patient cohort. Four patients in the overall enrolled cohort had rehospitalization and death recorded on the same day and were counted as death‐first. In the left atrial diameter cohort, the cumulative incidence of rehospitalization was 24.1% at 1 year and 36.2% at 3 years, compared with 12.2% and 21.3% for death‐first. Cumulative incidence curves by left atrial diameter tertile are shown in Figure S1.

Baseline characteristics of the 691‐patient left atrial diameter analysis cohort by study center are shown in Table S1. The NCGM cohort had more non‐paroxysmal AF, a more advanced New York Heart Association class, greater diuretic use, and a higher frequency of death‐first events, whereas the proportion with rehospitalization‐first was similar between cohorts.

### 2.2 Baseline Characteristics by LA Diameter

Larger LA diameter was associated with more non‐paroxysmal AF, lower LArs, younger age, and greater use of diuretics, whereas renal function, LVEF, and thromboembolic and bleeding‐risk scores were broadly similar across tertiles (Table 1).

**TABLE 1:** Baseline characteristics by left atrial diameter tertile in the left atrial diameter analysis cohort (*n* = 691).

Variable	LA small (*n* = 231)	LA mid (*n* = 249)	LA large (*n* = 211)	*p*
*N*	231	249	211	
Age, y	84.0 [76.0–88.5]	81.0 [73.0–88.0]	79.0 [70.0–85.0]	<0.001
Women, *n* (%)	112 (48)	119 (48)	103 (49)	0.975
Weight, kg	48.1 [40.3–57.3]	53.0 [43.7–62.0]	54.0 [44.0–64.2]	<0.001
Hemoglobin, g/dL	12.5 [10.8–14.1]	12.0 [10.4–13.6]	11.8 [10.1–13.8]	0.039
eGFR, mL/min/1.73 m^2^	46.6 [33.1–56.6]	44.8 [28.9–55.4]	40.7 [28.4–53.5]	0.078
LVEF, %	47.0 [35.3–61.4]	46.1 [34.4–58.1]	48.4 [31.9–59.7]	0.589
BNP, pg/mL (*n* = 320)	695.0 [422.5–1154.0]	573.0 [358.0–894.0]	691.5 [410.2–1103.2]	0.182
NT‐proBNP, pg/mL (*n* = 356)	4313.0 [2151.0–10766.0]	4889.0 [2138.0–8313.0]	3313.5 [2125.0–6076.8]	0.144
Natriuretic peptide (z) (*n* = 676)	0.1 [−0.5 to 0.8]	0.0 [−0.6 to 0.5]	−0.0 [−0.5 to 0.6]	0.546
NYHA class	3.0 [3.0–4.0]	3.0 [3.0–4.0]	3.0 [3.0–4.0]	0.383
HAS‐BLED score	2.0 [2.0–3.0]	2.0 [2.0–3.0]	2.0 [1.5–3.0]	0.936
CHA_2_DS_2_‐VASc score	5.0 [4.0–6.0]	5.0 [4.0–6.0]	5.0 [4.0–6.0]	0.312
History of stroke, *n* (%)	39 (17)	38 (15)	33 (16)	0.881
History of major bleeding, *n* (%)	6 (3)	11 (4)	13 (6)	0.185
Hypertension, *n* (%)	158 (68)	176 (71)	140 (66)	0.606
Diabetes mellitus, *n* (%)	70 (30)	69 (28)	64 (30)	0.771
Dyslipidemia, *n* (%)	81 (35)	98 (39)	77 (36)	0.611
Coronary artery disease, *n* (%) (*n* = 689)	54 (23)	66 (27)	50 (24)	0.701
Vascular disease, *n* (%)	62 (27)	70 (28)	59 (28)	0.945
COPD, *n* (%)	18 (8)	11 (4)	16 (8)	0.245
Non‐paroxysmal AF, *n* (%)	127 (55)	163 (65)	171 (81)	<0.001
Oral anticoagulant, *n* (%)	182 (79)	202 (81)	182 (86)	0.116
Direct oral anticoagulant, *n* (%)	135 (58)	131 (53)	96 (45)	0.025
Warfarin, *n* (%)	47 (20)	71 (29)	86 (41)	<0.001
Antiplatelet/NSAID, *n* (%)	59 (26)	68 (27)	71 (34)	0.143
Loop diuretics, *n* (%)	132 (57)	189 (76)	174 (82)	<0.001
MRA, *n* (%)	58 (25)	88 (35)	100 (47)	<0.001
β‐blockers, *n* (%)	141 (61)	164 (66)	139 (66)	0.458
ACEI/ARB/ARNI, *n* (%)	92 (40)	107 (43)	104 (49)	0.127
SGLT2 inhibitors, *n* (%)	4 (2)	7 (3)	4 (2)	0.682
LA diameter, mm	40.1 [36.5–42.5]	48.0 [46.0–50.0]	56.0 [53.0–60.0]	<0.001
LArs, % (*n* = 320)	9.8 [7.4–12.5]	9.2 [6.4–12.6]	8.2 [5.6–10.5]	<0.001
LAVI, mL/m^2^ (*n* = 320)	60.6 [47.3–75.6]	72.1 [56.7–90.2]	100.4 [77.5–137.2]	<0.001
E/e′ (*n* = 482)	14.8 [10.9–19.6]	16.7 [11.9–21.1]	17.5 [13.5–24.6]	<0.001
HF rehospitalization‐first, *n* (%)	48 (21)	64 (26)	77 (36)	<0.001
Death‐first, *n* (%)	46 (20)	37 (15)	35 (17)	0.331

*Notes*: Values are median [interquartile range] or *n* (%). LA diameter tertiles were defined as small ≤44.1 mm, mid >44.1 to ≤51.0 mm, and large >51.0 mm. First‐event counts classify rehospitalization and death recorded on the same day as death‐first. *P* values are descriptive and were not used for inferential interpretation.

Abbreviations: ACEI, angiotensin‐converting enzyme inhibitor; AF, atrial fibrillation; ARB, angiotensin receptor blocker; ARNI, angiotensin receptor‐neprilysin inhibitor; BNP, B‐type natriuretic peptide; COPD, chronic obstructive pulmonary disease; E/e′, ratio of early mitral inflow velocity to mitral annular early diastolic velocity; eGFR, estimated glomerular filtration rate; HF, heart failure; LA, left atrial; LArs, left atrial reservoir strain; LAVI, left atrial volume index; LVEF, left‐ventricular ejection fraction; MRA, mineralocorticoid receptor antagonist; NSAID, non‐steroidal anti‐inflammatory drug; NT‐proBNP, N‐terminal pro–B‐type natriuretic peptide; NYHA, New York Heart Association; SGLT2, sodium‐glucose cotransporter‐2.

### 2.3 LA Diameter and First HF Rehospitalization

Larger left atrial diameter was associated with a higher cumulative incidence of rehospitalization (Gray's *p* = 0.013) and remained associated with rehospitalization‐first after full adjustment (subdistribution hazard ratio [sHR] 1.025 per mm, 95% CI 1.011–1.039; *p* < 0.001), without attenuation across sequential adjustment (Table 2). Adjusted estimates were concordant at NCGM (sHR 1.271 per 10 mm, 95% CI 1.026–1.574; *p* = 0.028) and Toho (sHR 1.347, 95% CI 1.121–1.618; *p* = 0.002), with no interaction by study center (*p* = 0.930) (Table S2). The association was also consistent across alternative scaling, LVEF‐stratified analyses, additional covariate adjustment, alternative competing‐event definitions, and the 30‐day landmark analysis, and reclassifying same‐day events as rehospitalization‐first produced a similar estimate (Table S2).

**TABLE 2:** Left atrial diameter and heart failure rehospitalization versus death in the left atrial diameter analysis cohort.

Model/contrast	Estimate (95% CI)	*p*
Fine–Gray sHR, crude (per mm)	1.027 (1.014–1.041)	<0.001
Fine–Gray sHR, fully adjusted	1.025 (1.011–1.039)	<0.001
Cause‐specific HR, HF rehospitalization	1.023 (1.008–1.039)	0.003
Cause‐specific HR, death	0.982 (0.959–1.005)	0.120
Between‐cause divergence (Lunn–McNeil)	—	0.005

*Notes*: Fully adjusted for age, sex, eGFR, hemoglobin, LVEF, center, and natriuretic peptide (*n* = 676; 187 rehospitalization‐first and 111 death‐first events). Rehospitalization and death recorded on the same day were counted as death‐first.

Abbreviations: CI, confidence interval; eGFR, estimated glomerular filtration rate; HF, heart failure; HR, hazard ratio; LVEF, left‐ventricular ejection fraction; sHR, subdistribution hazard ratio.

### 2.4 Divergent Associations With Rehospitalization Versus Death

**FIGURE 1:**
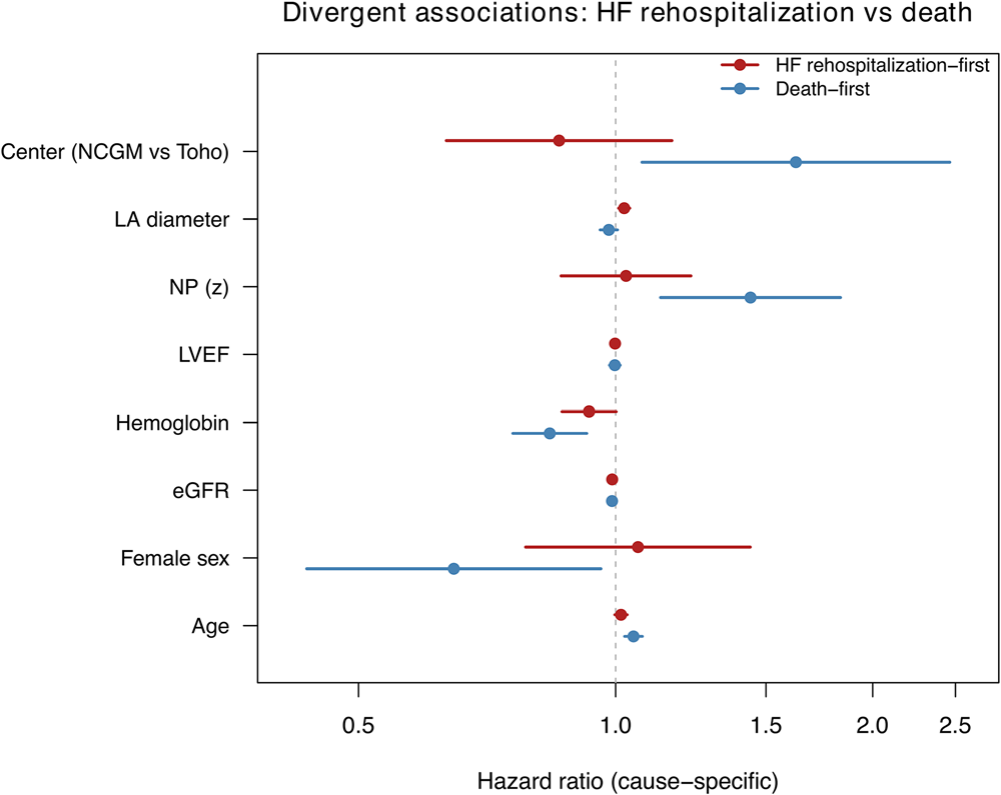
Divergent associations with rehospitalization and death. Cause‐specific hazard ratios compare HF rehospitalization‐first and death‐first in the left atrial diameter analysis cohort, with 95% confidence intervals for each variable. Same‐day rehospitalization and death were counted as death‐first. Toho University Ohashi Medical Center was the reference center. eGFR, estimated glomerular filtration rate; HF, heart failure; LA, left atrial; LVEF, left‐ventricular ejection fraction; NCGM, National Center for Global Health and Medicine; NP, natriuretic peptide.

LA diameter was not associated with death‐first (cause‐specific hazard ratio 0.982, 95% CI 0.959–1.005, *p* = 0.120), and its association differed between the two causes (Lunn–McNeil *p* = 0.005) (Figure 1). Age showed a differential association with death‐first (divergence *p* = 0.029), whereas the evidence for a differential association of natriuretic peptide was weaker (*p* = 0.064). eGFR and hemoglobin were associated with both outcomes without divergence.

### 2.5 Left Atrial Reservoir Strain and First and Recurrent Rehospitalization

**FIGURE 2:**
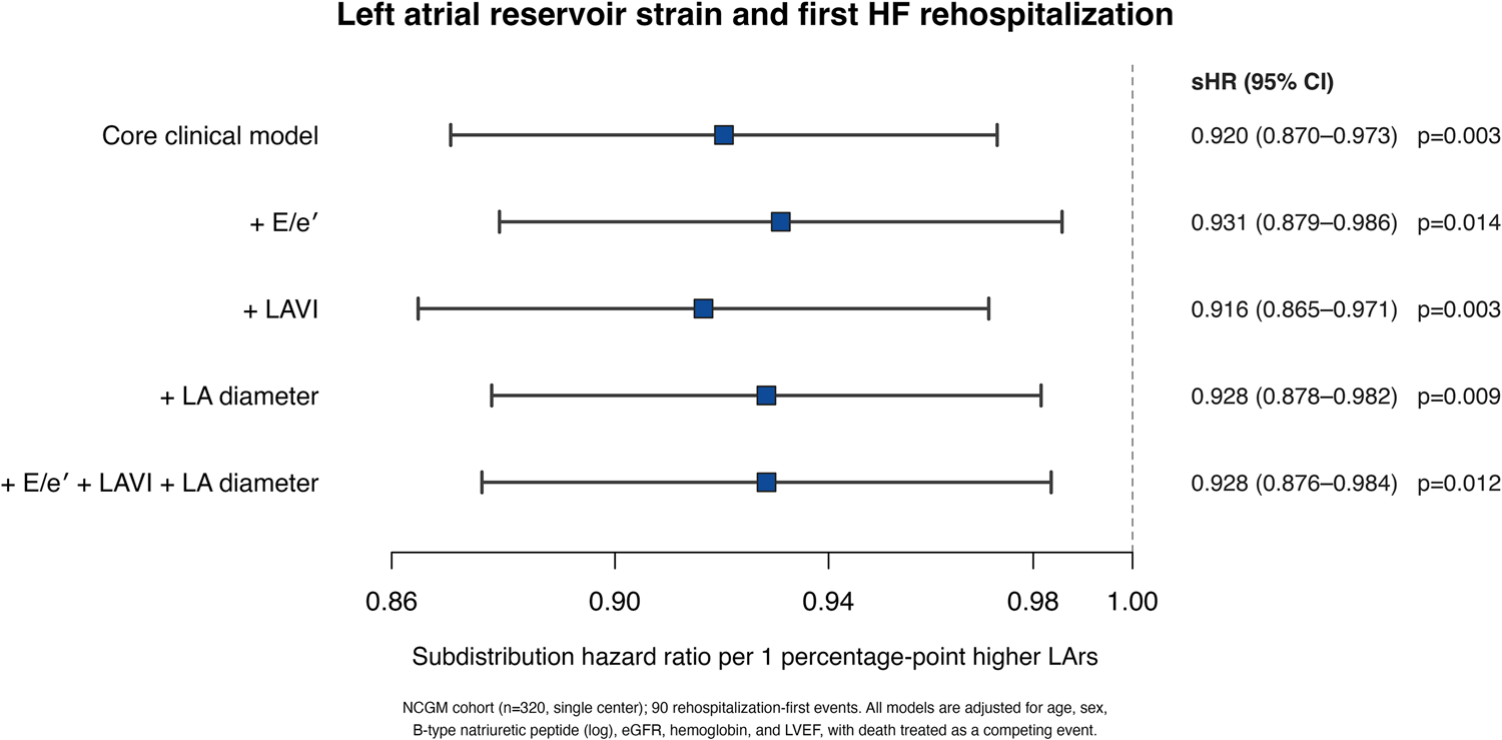
Left atrial reservoir strain and first heart failure rehospitalization across adjustment sets. Subdistribution hazard ratios with 95% confidence intervals show the association of LArs with first HF rehospitalization in the 320‐patient NCGM cohort, expressed per 1 percentage‐point higher LArs, with death treated as a competing event. Every model was adjusted for age, sex, B‐type natriuretic peptide (log), estimated glomerular filtration rate, hemoglobin, and left‐ventricular ejection fraction; each row adds the echocardiographic covariates shown. CI, confidence interval; E/e′, ratio of early mitral inflow velocity to mitral annular early diastolic velocity; HF, heart failure; LArs, left atrial reservoir strain; LAVI, left atrial volume index; NCGM, National Center for Global Health and Medicine; sHR, subdistribution hazard ratio.

Lower LArs was associated with first rehospitalization (adjusted sHR 0.920 per 1 percentage‐point increase, 95% CI 0.870–0.973, *p* = 0.003) but not with death‐first (*p* = 0.954). The estimate was essentially unchanged when E/e′ (sHR 0.931), LAVI (sHR 0.916), left atrial diameter (sHR 0.928, 95% CI 0.878–0.982, *p* = 0.009), or all three (sHR 0.928, 95% CI 0.876–0.984, *p* = 0.012) were added to the model (Figure 2; Table S3). For recurrent burden (169 readmissions), lower LArs was associated with a higher readmission rate (incidence rate ratio 0.877 per 1 percentage‐point increase, 95% CI 0.821–0.937, *p* < 0.001), whereas left atrial diameter and LAVI were not (Table 3).

**TABLE 3:** Left atrial indices and exploratory left atrial diameter–LArs categories in the NCGM cohort (*n* = 320).

Index or category (*n*)	First rehospitalization, sHR (95% CI)	Recurrent burden, IRR (95% CI)
Individual indices		
LArs, per 1 percentage point	0.920 (0.870–0.973), *p* = 0.003	0.877 (0.821–0.937), *p* < 0.001
LA diameter, per mm	1.024 (1.003–1.046), *p* = 0.028	1.020 (0.995–1.046), *p* = 0.123
LAVI, per mL/m^2^	1.000 (0.998–1.003), *p* = 0.760	0.999 (0.995–1.004), *p* = 0.809
Combined indices in one model		
LArs (with LA diameter)	0.928 (0.878–0.982), *p* = 0.009	—
LA diameter (with LArs)	1.018 (0.995–1.042), *p* = 0.130	—
LArs (with LAVI)	0.916 (0.865–0.971), *p* = 0.003	—
LAVI (with LArs)	0.999 (0.996–1.002), *p* = 0.510	—
Exploratory left atrial diameter–LArs categories		
Neither abnormality (73)	Reference	Reference
LA diameter only (64)	1.50 (0.75–3.03), *p* = 0.25	1.90 (0.86–4.19), *p* = 0.110
LArs only (78)	1.26 (0.62–2.58), *p* = 0.52	1.99 (0.92–4.31), *p* = 0.080
Both (105)	2.12 (1.16–3.85), *p* = 0.014	3.50 (1.75–6.99), *p* < 0.001

*Notes*: Analyses were performed in the single‐center NCGM cohort. Fine–Gray models for first rehospitalization were adjusted for age, sex, natriuretic peptide, eGFR, hemoglobin, and (for the individual and combined index models) LVEF; negative binomial models for recurrent burden were adjusted for age, sex, natriuretic peptide, eGFR, and hemoglobin, with log follow‐up time as an offset. The category analyses were exploratory and used cohort‐derived 365‐day competing‐risk cutoffs (left atrial diameter >48.6 mm and LArs <9.5%). Published reference thresholds classified 94%–96% of this cohort as abnormal and left no usable contrast (Table S5); median‐based categories were examined in Table S6. Recurrent burden reflects the total readmission count (169 readmissions) and is not a death‐adjusted recurrent‐event analysis. No category was associated with death‐first.

Abbreviations: CI, confidence interval; eGFR, estimated glomerular filtration rate; IRR, incidence rate ratio; LA, left atrial; LArs, left atrial reservoir strain; LAVI, left atrial volume index; LVEF, left‐ventricular ejection fraction; NCGM, National Center for Global Health and Medicine; sHR, subdistribution hazard ratio.

### 2.6 Comparison of Left Atrial Size Measures

In the same 320 patients, left atrial diameter was associated with first rehospitalization (sHR 1.024 per mm, 95% CI 1.003–1.046, *p* = 0.028), whereas LAVI was not (sHR 1.000 per mL/m^2^, 95% CI 0.998–1.003, *p* = 0.760) (Table 3). Expressed per standard deviation, the estimates were 1.245 (95% CI 1.024–1.514) for left atrial diameter and 1.022 (95% CI 0.885–1.181) for LAVI, so the same qualitative pattern was present once both measures were placed on a common scale. In a cause‐specific sensitivity analysis, neither measure showed clear evidence of departure from linearity (likelihood ratio *p* = 0.066 for left atrial diameter and *p* = 0.248 for LAVI), and the two were only moderately correlated (Spearman rho 0.555). Correlations between LArs and the two size measures were weak and variance inflation factors were low, so collinearity does not explain these results (Table S4).

### 2.7 Exploratory Left Atrial Diameter–LArs Categories

**FIGURE 3:**
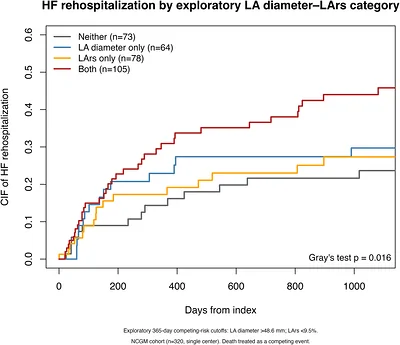
Heart failure rehospitalization by exploratory left atrial diameter–LArs category. Cumulative incidence functions compare HF rehospitalization across the four exploratory categories in the 320‐patient NCGM cohort, with death treated as a competing event. Categories were defined by the cohort‐derived 365‐day competing‐risk cutoffs of left atrial diameter >48.6 mm and LArs <9.5%, and are labeled neither, LA diameter only, LArs only, and both. CIF, cumulative incidence function; HF, heart failure; LA, left atrial; LArs, left atrial reservoir strain; NCGM, National Center for Global Health and Medicine.

Published reference thresholds classified 94%–96% of patients as abnormal and did not provide a usable contrast (Table S5). At 365 days, the exploratory cohort‐derived competing‐risk cutoffs were 48.6 mm for left atrial diameter (sensitivity 66.2%, specificity 52.3%; AUC 0.579) and 9.5% for LArs (sensitivity 52.7%, specificity 63.7%; AUC 0.573). Using these cutoffs, only the category with both an enlarged left atrial diameter and a low LArs was associated with first rehospitalization (adjusted sHR 2.12, 95% CI 1.16–3.85, *p* = 0.014) and with recurrent readmission burden (incidence rate ratio 3.50, 95% CI 1.75–6.99, *p* < 0.001); neither single‐abnormality category reached significance for either outcome (Figure 3; Figure S2; Table 3). No category was associated with death‐first; median‐based categories and a tertile‐extreme definition were examined in sensitivity analyses (Table S6).

### 2.8 Incremental Discrimination and Model Checks

Addition of left atrial diameter, LArs, LAVI, or their combination produced only small, non‐significant changes in the time‐dependent AUC and Brier score; the largest gain was +0.044 at 3 years for left atrial diameter plus LArs (*p* = 0.115) (Table S7). The principal associations were materially unchanged after adjustment for body mass index and significant MR and when chronic kidney disease replaced continuous eGFR, and both remained significant (Table S8). Model‐assumption checks showed no important violations (Table S6).

## 3 Discussion

In patients hospitalized for AHF with AF, lower left atrial reservoir strain was associated with both the first HF rehospitalization and the burden of recurrent readmission, and that association persisted when E/e′, LAVI, and left atrial diameter were added to the model. In the larger cohort, left atrial diameter was associated with HF rehospitalization‐first rather than death‐first. This was not simply a matter of larger atria marking sicker patients. Age was more closely associated with death‐first, whereas evidence for a differential association of natriuretic peptide was weaker, and LA diameter remained associated with rehospitalization after sequential clinical adjustment. Such a separation is biologically plausible. Survival and readmission after a heart‐failure hospitalization can move independently [17], and the markers of readmission and death differ across the ejection‐fraction spectrum in Asian populations [18]. In our cohort, too, HF rehospitalization was more frequent than death‐first. Readmission and death after discharge therefore behave as different outcomes in this population, and a competing first‐event framework brings out markers of recurrent congestion risk that mortality‐centered analyses can miss [3, 19].

The contrast between left atrial diameter and LAVI needs a careful reading, because LAVI is the recommended volumetric measure of left atrial size [5] and yet was associated with neither outcome here. Several candidate explanations can be examined in these data. The pattern was not produced by the measurement scale: expressed per standard deviation, the association with first rehospitalization persisted for diameter (1.245, 95% CI 1.024–1.514) and remained absent for LAVI (1.022, 95% CI 0.885–1.181), so the same qualitative contrast was present once both measures were placed on a common scale. This side‐by‐side comparison is descriptive, and the two coefficients were not formally tested against each other. Both indices were measured in the same 320 patients and were analyzed in separate Fine–Gray models with identical covariate adjustment and the same 90 rehospitalization‐first events, so a simple imbalance in sample size or event count is an unlikely explanation, with the qualification that equal measurement precision for the two indices is not thereby demonstrated. In a cause‐specific sensitivity analysis comparing a spline term with the linear term, neither index showed clear evidence of non‐linearity, which does not exclude non‐linearity in the subdistribution models. What these data cannot separate is measurement‐related discordance from residual confounding. The anteroposterior diameter is a single linear dimension recorded from a standard parasternal view, whereas LAVI is planimetered from apical views and is sensitive to foreshortening, to beat‐to‐beat variation during AF, and to loading conditions at an acute admission; in addition, 96% of this cohort already exceeded the guideline volume threshold, so the variation that remained in LAVI may carry proportionally more measurement noise. A genuinely different biological association remains possible but is not demonstrated. We therefore read the diameter finding as a routinely available marker within this cohort, not as evidence that left atrial structural remodeling in general predicts rehospitalization and not as evidence that diameter should replace LAVI.

Within that boundary, atrial size and reservoir strain still appear to mark overlapping but distinct aspects of one substrate [4]. Left atrial enlargement reflects chronic remodeling under a sustained filling‐pressure load, so atrial size may reflect cumulative remodeling related to AF, fibrosis, and repeated pressure and volume loading rather than the filling pressure present at the time of imaging; left atrial diameter in this setting should therefore not be read as a surrogate for filling pressure, and we do not claim that it measures one. Reservoir strain, by contrast, reflects how the atrium deforms. In the exploratory cutoff analyses, only the category with both an enlarged diameter and a low LArs was associated with first rehospitalization and with recurrent readmission burden, whereas neither single‐abnormality category reached significance; the association of diameter with first rehospitalization was attenuated once LArs entered the model, whereas LArs was not attenuated by diameter. The weak size–strain correlations we observed (rho −0.18 for left atrial diameter and −0.25 for LAVI) also temper the purely geometric explanation that a larger atrium must deform less: geometry contributes, but it leaves most of the variance in LArs unexplained.

LA enlargement reflects long‐standing exposure to elevated filling pressure, whereas reduced reservoir strain is more closely tied to current filling pressure [6]; accordingly, low LA reservoir strain has been linked to adverse outcomes across HF and AF cohorts [20, 21, 22, 23], and in our data reduced LArs may signal persistent elevation in filling pressure and a tendency toward recurrent congestion. LArs was measured during acute decompensation and may therefore reflect both transient loading conditions and underlying atrial dysfunction. Its association with first rehospitalization persisted after adjustment for E/e′, LAVI, and left atrial diameter, but the present study cannot distinguish an acute hemodynamic marker from a fixed atrial substrate. Serial measurements after decongestion would be required to separate these possibilities.

Incremental predictive performance was limited, and even meta‐analytic reclassification gains for LA strain are modest [20]; these findings should therefore be interpreted as associations rather than as evidence for a clinical prediction tool. LA diameter is readily available on routine echocardiography, and LArs, where it is measured, characterizes a functional substrate associated with recurrent decompensation.

Several limitations should be acknowledged. This retrospective, two‐center observational study used complete‐case analyses without imputation, and residual confounding cannot be excluded. Although the site‐specific estimates were concordant and the interaction was not significant, measured and unmeasured between‐center differences may still limit the generalizability of the strain‐related findings. LAVI was not associated with either outcome in the NCGM cohort, and the association of left atrial diameter was attenuated after adjustment for LArs; the findings therefore do not demonstrate superiority of left atrial diameter over LAVI or imply that all structural left atrial measures are associated with rehospitalization. The LArs findings remain single‐center and may not generalize to other settings. The threshold‐based categories were derived and evaluated in the same cohort, were exploratory, and require external validation. Individual readmission dates were unavailable, so recurrent burden was modeled as a count rather than with death‐adjusted recurrent‐event methods such as joint‐frailty models [24]; event timing and dependence on terminal death were therefore not fully addressed. Multiple secondary and sensitivity analyses were conducted without formal multiplicity adjustment, so some findings may reflect chance. LArs was measured during acute decompensation from a single cardiac cycle in each view, and AF‐related beat‐to‐beat variability, abnormal loading conditions, image quality, chamber enlargement, lateral‐wall resolution, and rapid ventricular response may affect tracking. The prior protocol showed good cross‐view heart‐rate agreement and intra‐observer reproducibility, and two apical views were averaged within the standardized strain framework [25]; these features reduce, but do not eliminate, AF‐related measurement variability. Because strain analysis was performed by a single observer, inter‐observer reproducibility could not be evaluated; beat‐to‐beat reproducibility was also not assessed.

## 4 Conclusion

Lower LArs was associated with both first HF rehospitalization and recurrent readmission burden, and remained associated when E/e′, LAVI, and left atrial diameter were added to the model. LA diameter was associated with first HF rehospitalization in the larger cohort, whereas LAVI was not associated with either first rehospitalization or recurrent readmission burden, so these results should not be read as evidence for left atrial structural remodeling in general. None of the left atrial indices significantly improved discrimination, and the cohort‐derived cutoff analyses were exploratory and require external validation. Where reservoir strain is available, it characterizes a functional aspect of the LA that marks rehospitalization burden in patients admitted with AHF and AF.

## Conflicts of Interest

The authors declare no conflicts of interest.

## Funding

This research received no grant from any funding agency in the public, commercial or not‐for‐profit sectors.

## Ethics Statement

The study was approved by the Institutional Review Board of the National Center for Global Health and Medicine (approval number JIHS‐S‐005141‐00) and was conducted in accordance with the Declaration of Helsinki. The requirement for written informed consent was waived because only anonymized retrospective data were used.

## Supporting information




**Supporting information**: echo70616‐sup‐0001‐SuppMat.docx

## Data Availability

The data underlying this article cannot be shared publicly because of privacy and ethical restrictions for anonymized clinical registry data. Reasonable inquiries may be directed to the corresponding author and will require applicable institutional approval.
