# Supplementary material for: Left Atrial Reservoir Strain and Heart Failure Rehospitalization Burden in Acute Heart Failure With Atrial Fibrillation
Source: Echocardiography. 2026 Sep 4;43(9):e70616. doi: 10.1111/echo.70616 (PMC13544991; doi:10.1111/echo.70616)
Supplement: Supplementary file 1 [file ECHO-43-e70616-s001.docx]

**Supporting Information**

Left Atrial Reservoir Strain and Heart Failure Rehospitalization Burden in Acute Heart Failure With Atrial Fibrillation

Table S1. Baseline characteristics of the left atrial diameter analysis cohort by study center.

| **Variable** | **NCGM cohort (n=320)** | **Toho cohort (n=371)** | **SMD** |
| --- | --- | --- | --- |
| Age, y | 81.0 [72.8-87.0] | 81.0 [73.0-87.0] | -0.012 |
| Women, n (%) | 163 (50.9) | 171 (46.1) | 0.097 |
| Weight, kg | 49.5 [42.0-60.2] | 51.5 [43.7-61.8] | -0.074 |
| Hemoglobin, g/dL | 12.3 [10.4-13.9] | 11.9 [10.3-13.9] | 0.055 |
| eGFR, mL/min/1.73 m² | 46.1 [31.5-59.1] | 42.3 [28.1-53.5] | 0.202 |
| LVEF, % | 45.1 [34.7-55.4] | 49.1 [32.5-63.9] | -0.168 |
| NYHA class | 4.0 [3.0-4.0] | 3.0 [2.0-3.0] | 0.780 |
| LA diameter, mm | 49.0 [43.3-54.0] | 47.0 [42.0-51.0] | 0.242 |
| Non-paroxysmal AF, n (%) | 284 (88.8) | 177 (47.7) | 0.982 |
| Hypertension, n (%) | 236 (73.8) | 238 (64.2) | 0.209 |
| Diabetes mellitus, n (%) | 96 (30.0) | 107 (28.8) | 0.025 |
| Coronary artery disease, n (%) | 71 (22.2) | 99 (26.8) | -0.108 |
| Oral anticoagulant, n (%) | 269 (84.1) | 297 (80.1) | 0.105 |
| Direct oral anticoagulant, n (%) | 129 (40.3) | 233 (62.8) | -0.462 |
| Warfarin, n (%) | 140 (43.8) | 64 (17.3) | 0.601 |
| Antiplatelet/NSAID, n (%) | 95 (29.7) | 103 (27.8) | 0.043 |
| ACEI/ARB, n (%) | 131 (40.9) | 172 (46.4) | -0.110 |
| β-blocker, n (%) | 235 (73.4) | 209 (56.3) | 0.364 |
| MRA, n (%) | 146 (45.6) | 100 (27.0) | 0.396 |
| Loop diuretic, n (%) | 287 (89.7) | 208 (56.1) | 0.817 |
| SGLT2 inhibitor, n (%) | 6 (1.9) | 9 (2.4) | -0.038 |
| BNP measured, n (%) | 320 (100.0) | 0 (0.0) | — |
| NT-proBNP measured, n (%) | 0 (0.0) | 356 (96.0) | — |
| Follow-up, days | 527.5 [83.0-1519.5] | 494.0 [89.0-781.0] | 0.485 |
| HF rehospitalization-first, n (%) | 90 (28.1) | 99 (26.7) | 0.032 |
| Death-first, n (%) | 72 (22.5) | 46 (12.4) | 0.269 |

Values are median [interquartile range] or n (%). Standardized mean differences are shown; p values are not reported because this comparison is descriptive. This table includes the 691 patients with an available left atrial diameter measurement (320 at NCGM and 371 at Toho). The NCGM cohort had more non-paroxysmal AF, a higher NYHA class, and more intensive diuretic therapy. LAVI and LArs were available only in the NCGM cohort; LArs was retrospectively measured offline from routinely acquired apical 2- and 4-chamber images. Natriuretic peptide was measured as BNP at NCGM and as NT-proBNP at Toho. ACEI, angiotensin-converting enzyme inhibitor; AF, atrial fibrillation; ARB, angiotensin receptor blocker; BNP, B-type natriuretic peptide; eGFR, estimated glomerular filtration rate; HF, heart failure; LA, left atrial; LArs, left atrial reservoir strain; LAVI, left atrial volume index; LVEF, left-ventricular ejection fraction; MRA, mineralocorticoid receptor antagonist; NCGM, National Center for Global Health and Medicine; NSAID, non-steroidal anti-inflammatory drug; NT-proBNP, N-terminal pro–B-type natriuretic peptide; NYHA, New York Heart Association; SGLT2, sodium-glucose cotransporter-2; SMD, standardized mean difference.

Table S2. Sensitivity analyses for left atrial diameter and heart failure rehospitalization in the left atrial diameter analysis cohort (Fine–Gray models; same-day events classified as death-first unless stated otherwise).

| **Analysis** | **Estimate (95% CI)** | **p** |
| --- | --- | --- |
| Per 10 mm | sHR 1.282 (1.118–1.470) | <0.001 |
| LA diameter × center interaction | — | 0.930 |
| LA diameter × LVEF interaction | — | 0.670 |
| HFpEF (LVEF ≥50%) | sHR 1.026 (1.008–1.045) | 0.005 |
| LVEF <50% | sHR 1.023 (1.001–1.046) | 0.044 |
| + antiplatelet/NSAID + oral anticoagulant | sHR 1.021 (1.007–1.036) | 0.004 |
| + HF therapies (ACEI/ARB, β-blocker, MRA, SGLT2i, diuretic) | sHR 1.023 (1.008–1.038) | 0.002 |
| + hypertension + diabetes | sHR 1.025 (1.011–1.040) | <0.001 |
| Chronic kidney disease (eGFR <60) replacing continuous eGFR | sHR 1.025 (1.011–1.039) | <0.001 |
| Cardiovascular death as competing event | sHR 1.025 (1.011–1.039) | <0.001 |
| 30-day landmark | sHR 1.028 (1.013–1.042) | <0.001 |
| Center-stratified: NCGM (per 10 mm) | sHR 1.271 (1.026–1.574) | 0.028 |
| Center-stratified: Toho (per 10 mm) | sHR 1.347 (1.121–1.618) | 0.002 |
| Same-day events reclassified as rehospitalization-first | sHR 1.024 (1.010–1.038) | <0.001 |

All models are adjusted for age, sex, eGFR, hemoglobin, LVEF, center, and natriuretic peptide unless the variable is the subject of the analysis. ACEI, angiotensin-converting enzyme inhibitor; ARB, angiotensin receptor blocker; CI, confidence interval; eGFR, estimated glomerular filtration rate; HF, heart failure; HFpEF, HF with preserved ejection fraction; LA, left atrial; LVEF, left-ventricular ejection fraction; MRA, mineralocorticoid receptor antagonist; NCGM, National Center for Global Health and Medicine; NSAID, non-steroidal anti-inflammatory drug; SGLT2i, sodium-glucose cotransporter-2 inhibitor; sHR, subdistribution hazard ratio.

Table S3. Left atrial reservoir strain independent of diastolic, volume, and diameter indices (NCGM cohort, n=320).

| **Model** | **Estimate (95% CI)** | **p** |
| --- | --- | --- |
| Association of LArs with HF rehospitalization-first, adjusted | sHR 0.920 (0.870–0.973) | 0.003 |
| Additionally adjusted for E/e′ | sHR 0.931 (0.879–0.986) | 0.014 |
| Additionally adjusted for LAVI | sHR 0.916 (0.865–0.971) | 0.003 |
| Additionally adjusted for left atrial diameter | sHR 0.928 (0.878–0.982) | 0.009 |
| Additionally adjusted for E/e′ and LAVI | sHR 0.925 (0.872–0.981) | 0.010 |
| Additionally adjusted for E/e′, LAVI, and left atrial diameter | sHR 0.928 (0.876–0.984) | 0.012 |

Adjusted for age, sex, B-type natriuretic peptide (log), eGFR, hemoglobin, and LVEF. CI, confidence interval; E/e′, ratio of early mitral inflow velocity to mitral annular early diastolic velocity; eGFR, estimated glomerular filtration rate; HF, heart failure; LA, left atrial; LArs, left atrial reservoir strain; LAVI, left atrial volume index; LVEF, left-ventricular ejection fraction; NCGM, National Center for Global Health and Medicine; sHR, subdistribution hazard ratio.

Table S4. Comparison of left atrial size and reservoir strain measures (NCGM cohort, n=320).

| **Analysis** | **Statistic** | **p** |
| --- | --- | --- |
| LA diameter vs LArs | Spearman rho −0.181 | 0.001 |
| LAVI vs LArs | Spearman rho −0.253 | <0.001 |
| LA diameter vs LAVI | Spearman rho 0.555 | <0.001 |
| Variance inflation factor, LA diameter (in the model containing LA diameter and LArs) | 1.11 | — |
| Variance inflation factor, LArs (in the model containing LA diameter and LArs) | 1.15 | — |
| Variance inflation factor, LAVI (in the model containing LAVI and LArs) | 1.16 | — |
| Variance inflation factor, LArs (in the model containing LAVI and LArs) | 1.16 | — |
| Adjusted sHR per 1 SD, left atrial diameter (9.15 mm) | 1.245 (1.024–1.514) | 0.028 |
| Adjusted sHR per 1 SD, LAVI (56.12 mL/m²) | 1.022 (0.885–1.181) | 0.760 |
| Adjusted sHR per 1 SD, LArs (4.68 percentage points) | 0.678 (0.523–0.879) | 0.003 |
| Departure from linearity, left atrial diameter | Likelihood ratio test | 0.066 |
| Departure from linearity, LAVI | Likelihood ratio test | 0.248 |

Variance inflation factors were computed on the same complete-case design as the corresponding Fine–Gray models. All values are far below conventional thresholds for collinearity. Per-standard-deviation estimates are Fine–Gray subdistribution hazard ratios for first heart failure rehospitalization, with 95% confidence intervals in parentheses, adjusted for age, sex, B-type natriuretic peptide (log), eGFR, hemoglobin, and left-ventricular ejection fraction. Departure from linearity was assessed by likelihood ratio comparing a natural spline (3 degrees of freedom) with the linear term in the cause-specific model; the corresponding test for LArs is shown in Table S6. CI, confidence interval; eGFR, estimated glomerular filtration rate; LA, left atrial; LArs, left atrial reservoir strain; LAVI, left atrial volume index; NCGM, National Center for Global Health and Medicine; SD, standard deviation; sHR, subdistribution hazard ratio.

Table S5. Published reference-threshold categories of left atrial volume index and reservoir strain (NCGM cohort, n=320).

| **Category** | **n** | **HF rehospitalization-first, n** | **Death-first, n** |
| --- | --- | --- | --- |
| Neither (LAVI ≤34 mL/m² and LArs ≥18%) | 2 | 1 | 0 |
| LAVI only (LAVI >34 mL/m², LArs ≥18%) | 17 | 1 | 4 |
| LArs only (LAVI ≤34 mL/m², LArs <18%) | 11 | 1 | 5 |
| Both (LAVI >34 mL/m², LArs <18%) | 290 | 87 | 63 |

The published reference thresholds were LArs <18% for impaired reservoir function and LAVI >34 mL/m² for an enlarged left atrium. In this cohort 301 of 320 patients (94.1%) had LArs <18% and 307 (95.9%) had LAVI >34 mL/m². Because three of the four categories contained fewer than 30 patients and fewer than five rehospitalization-first events, adjusted estimates were not calculated. HF, heart failure; LArs, left atrial reservoir strain; LAVI, left atrial volume index; NCGM, National Center for Global Health and Medicine.

Table S6. Model-assumption checks and category cutoff sensitivity analyses.

| **Check** | **Statistic** | **p** |
| --- | --- | --- |
| Proportional hazards — LA diameter, cause-specific HF model (LA-diameter cohort) | Global Schoenfeld test | 0.099 |
| LArs non-linearity (natural spline vs linear, cause-specific HF) | Likelihood ratio test | 0.681 |
| Both abnormal, largest LA diameter tertile with lowest LArs tertile | sHR 1.856 (1.126–3.059) | 0.015 |
| Median-based categories: LA diameter only | sHR 1.50 (0.79–2.86) | 0.21 |
| Median-based categories: LArs only | sHR 1.42 (0.71–2.81) | 0.32 |
| Median-based categories: both abnormal | sHR 2.27 (1.26–4.09) | 0.006 |
| Median-based categories, recurrent: LA diameter only | IRR 1.92 (0.93–4.00) | 0.080 |
| Median-based categories, recurrent: LArs only | IRR 2.57 (1.24–5.33) | 0.011 |
| Median-based categories, recurrent: both abnormal | IRR 4.43 (2.25–8.72) | <0.001 |

Median-based categories were defined as left atrial diameter >49.0 mm and LArs <8.775% and were examined as sensitivity analyses. The main-text categories used the cohort-derived 365-day competing-risk cutoffs of left atrial diameter >48.6 mm and LArs <9.5%. The tertile-extreme category was defined as left atrial diameter in the top third and LArs in the bottom third of the NCGM cohort (n=46, of whom 21 had a rehospitalization-first event). Category models were adjusted for age, sex, B-type natriuretic peptide (log), eGFR, and hemoglobin. Values in parentheses are 95% CIs. CI, confidence interval; eGFR, estimated glomerular filtration rate; HF, heart failure; IRR, incidence rate ratio; LA, left atrial; LArs, left atrial reservoir strain; NCGM, National Center for Global Health and Medicine; sHR, subdistribution hazard ratio.

Table S7. Incremental discrimination of left atrial indices (time-dependent AUC and Brier score).

| **Model** | **AUC 1 y (95% CI)** | **AUC 3 y (95% CI)** | **ΔAUC 1 y / 3 y** | **p 1 y / 3 y** |
| --- | --- | --- | --- | --- |
| LA-diameter complete-case cohort (n=676): clinical model | 0.621 (0.568–0.675) | 0.657 (0.600–0.713) | — | — |
| LA-diameter complete-case cohort: clinical model plus LA diameter | 0.648 (0.594–0.702) | 0.673 (0.617–0.729) | +0.026 / +0.016 | 0.142 / 0.396 |
| NCGM cohort (n=320): clinical model | 0.654 (0.570–0.738) | 0.650 (0.574–0.725) | — | — |
| NCGM cohort: clinical model plus LA diameter | 0.672 (0.589–0.755) | 0.669 (0.594–0.744) | +0.018 / +0.020 | 0.439 / 0.338 |
| NCGM cohort: clinical model plus LAVI | 0.655 (0.571–0.739) | 0.650 (0.575–0.726) | +0.001 / +0.001 | 0.845 / 0.835 |
| NCGM cohort: clinical model plus LArs | 0.663 (0.580–0.746) | 0.684 (0.611–0.757) | +0.009 / +0.035 | 0.745 / 0.169 |
| NCGM cohort: clinical model plus LA diameter and LArs | 0.675 (0.592–0.759) | 0.694 (0.620–0.767) | +0.021 / +0.044 | 0.484 / 0.115 |
| NCGM cohort: clinical model plus LAVI and LArs | 0.663 (0.580–0.746) | 0.683 (0.610–0.756) | +0.009 / +0.034 | 0.730 / 0.183 |

Brier scores, left atrial diameter complete-case cohort: clinical 0.179 (1 y) and 0.219 (3 y) versus the clinical model plus left atrial diameter 0.176 and 0.215 (p=0.112 and 0.188). Brier scores, NCGM cohort: clinical 0.164 and 0.206 versus the clinical model plus left atrial diameter and LArs 0.160 and 0.196 (p=0.256 and 0.208). The clinical model comprises age, sex, eGFR, hemoglobin, LVEF, and natriuretic peptide, plus center in the left atrial diameter cohort. Models were fitted with Fine–Gray regression and scored with inverse-probability-of-censoring weighting at 1 and 3 years on the same complete-case data (90 rehospitalization-first events in the NCGM cohort). No comparison reached statistical significance. AUC, area under the time-dependent receiver-operating-characteristic curve; CI, confidence interval; eGFR, estimated glomerular filtration rate; LA, left atrial; LArs, left atrial reservoir strain; LAVI, left atrial volume index; LVEF, left-ventricular ejection fraction; NCGM, National Center for Global Health and Medicine.

Table S8. Additional covariate sensitivity analyses in the NCGM cohort.

| **Model** | **n** | **Events** | **Estimate (95% CI)** | **p** |
| --- | --- | --- | --- | --- |
| LA diameter, core model | 320 | 90 | sHR 1.024 (1.003–1.046) | 0.028 |
| LA diameter + BMI | 320 | 90 | sHR 1.023 (1.001–1.046) | 0.038 |
| LA diameter + significant MR | 320 | 90 | sHR 1.025 (1.004–1.047) | 0.019 |
| LA diameter + BMI + significant MR | 320 | 90 | sHR 1.024 (1.003–1.046) | 0.025 |
| LArs, core model | 320 | 90 | sHR 0.920 (0.870–0.973) | 0.003 |
| LArs + BMI | 320 | 90 | sHR 0.922 (0.872–0.976) | 0.005 |
| LArs + significant MR | 320 | 90 | sHR 0.923 (0.874–0.975) | 0.004 |
| LArs + BMI + significant MR | 320 | 90 | sHR 0.925 (0.875–0.978) | 0.006 |
| LArs, CKD replacing continuous eGFR | 320 | 90 | sHR 0.920 (0.871–0.972) | 0.003 |

Models are Fine–Gray subdistribution hazard models for first heart failure rehospitalization, adjusted for the core covariates (age, sex, B-type natriuretic peptide [log], eGFR, hemoglobin, and left-ventricular ejection fraction), except that the CKD sensitivity model replaced continuous eGFR with CKD. Significant MR was defined as moderate or greater and was present in 100 of 320 patients; the remainder were classified as without significant MR. Body mass index was available in all 320 patients. BMI, body mass index; CI, confidence interval; CKD, chronic kidney disease; eGFR, estimated glomerular filtration rate; LA, left atrial; LArs, left atrial reservoir strain; MR, mitral regurgitation; NCGM, National Center for Global Health and Medicine; sHR, subdistribution hazard ratio.

Figure S1. Cumulative incidence of heart failure rehospitalization by left atrial diameter tertile.

Cumulative incidence functions compare left atrial diameter tertiles in the left atrial diameter analysis cohort (n=691), with death treated as a competing event. Follow-up was truncated at 1095 days. CIF, cumulative incidence function; HF, heart failure; LA, left atrial.

Figure S2. First and recurrent heart failure burden by exploratory left atrial diameter–LArs category.

The left panel shows adjusted subdistribution hazard ratios for first HF rehospitalization and the right panel adjusted incidence rate ratios for recurrent readmission burden, with 95% confidence intervals and the category with neither abnormality as the reference. Categories were defined by the cohort-derived 365-day competing-risk cutoffs of LA diameter >48.6 mm and LArs <9.5%, and are labelled neither, LA diameter only, LArs only, and both, in the 320-patient NCGM cohort. CI, confidence interval; HF, heart failure; IRR, incidence rate ratio; LA, left atrial; LArs, left atrial reservoir strain; NCGM, National Center for Global Health and Medicine; sHR, subdistribution hazard ratio.
